# Commentary: Lessons from the Analysis of Non-human Primates for Understanding Human Aging and Neurodegenerative Diseases

**DOI:** 10.3389/fnhum.2016.00033

**Published:** 2016-02-02

**Authors:** Andre Menache, Anne Beuter

**Affiliations:** ^1^Antidote Europe, Perpignan, France; ^2^Bordeaux Polytechnic Institute, Bordeaux, France

**Keywords:** non-human primate, Parkinson’s disease, animal model, prediction, neuromodulation

We agree with Verdier et al. (2015) that the use of animals may be scientifically viable in some areas of research, but we feel that the animal model is not sufficiently evidence-based to be considered as a valid predictive modality for the study of human disease. Our view is supported by empirical evidence (the lack of predictive ability of preclinical animal tests in the field of regulatory toxicology), by complexity theory (the inability of one evolved complex system to predict the response of a different evolved complex system to a given substance or stimulus) and by evolutionary biology (humans and other mammals share many of the same genes, but gene regulation can vary widely between different mammalian species) (Shanks and Greek, 2009; Godlee, 2014).

Therefore, we view as somewhat contradictory some of the arguments presented in relation to the following concluding remark proposed by the authors: “NHPs should only be used when no other suitable method is available to fill the gap of our knowledge” (page 4). For example, in the section entitled “The MPTP (1-methyl-4-phenyl-1,2,5,6-tetrahydropyridine) NHP models of neurodegeneration and the renaissance of Parkinson’s disease (PD) research,” the authors write: “Importantly, the MPTP NHP models allowed the identification of the neural circuits affected in Parkinson’s disease, specifically the role of excessive activity in the subthalamic nuclei (Bergman et al., 1990), leading to the development of ablative procedures (Baron et al., 2002) and of deep-brain stimulation (DBS) of these nuclei (DeLong and Benabid, 2014). These latter experiments (*sic*) would not have been developed without knowing the physiology of the basal ganglia in non-human primates (Baron et al., 2002).”

The MPTP primate model of PD proposed by Burns et al. (1983) followed the observation of a case of parkinsonism occurring after the self-administration by a 23-year-old man of an illicit synthetic drug [1-methyl-4-phenyl-4-propionoxy-piperidine (MPPP)]. In parallel, Langston et al. (1983) reported marked parkinsonism in four persons after using this drug intravenously. It was observed that this drug damaged the aminergic neurons of the substantia nigra.

Following this discovery, numerous studies based on the MPTP primate model were published over the last 25 years using various administration regimens. However, this model is not without limitations as indicated by Fox and Brotchie (2010) (a reference cited by the authors), who write that “Despite these obvious successes, limitations still exist in the model, particularly when considering underlying mechanisms of disease progression; thus, it appears difficult to reliably use acute toxin administration to replicate a chronic progressive disorder and provide consistent evidence of Lewy-like bodies.” Indeed as indicated by Halliday et al. (2009), inclusion of Lewy body-like formation is not prevalent in the MPTP monkey model. However, treatment of the NHP with l-DOPA induced involuntary movements resembling the phenomenology of human dyskinesias (Johnston and Fox, 2015).

Furthermore, long before the MPTP model existed, basic concepts of deep-brain stimulation had been developed in the 1960s. Jasper (1966) used microelectrodes to record electrical activity from single nerve fibers in the thalamus of patients with PD and showed that stimulation of the junction of the ventralis lateralis and the ventralis posterior nuclei of the thalamus stopped their tremor. Early surgical interventions for PD in human patients were attempted throughout the twentieth century by performing extirpations, clipping or lesions in multiple locations from the cortex to the spinal cord. Later, the number of surgeries decreased after l-DOPA became available at the end of the 60s. This number started to increase again after the presence of severe side effects associated with l-DOPA administration was reported. However, the proposed surgeries used reversible chronic electrical stimulation of various brain nuclei. Benabid et al. (1987) reported tremor reduction using deep-brain stimulation of the ventrointermediate nucleus of the thalamus in patients with PD. This report was preceded by the work of Bechtereva et al. (1975), and thus several years before the first MPTP study.

More recently, Drouot et al. (2004) observed clinical improvements in akinesia and bradykinesia following electrical stimulation of the motor cortex of baboons, along with a normalization of firing rates and synchronization in the subthalamic nucleus and internal globus pallidus. The following clinical trial in patients with PD (http://clinicaltrials.gov/show/NCT00159172) did not demonstrate similar improvements and was interrupted. So, it is not clear to us what the authors are referring to when they refer to a “renaissance of Parkinson’s disease research.”

Computational (*in silico*) models can contribute to explore the effects of DBS in PD, and there are today four main modeling approaches including single-compartment spiking neuron models, phase oscillator models, multicompartmental spiking neuron models, and more recently, neural field models. Each type of model has its own assumptions, strengths, limitations, and advocates (for details, see Modolo et al., 2010). In the human brain, compensatory mechanisms and plasticity are fundamental issues and biologically realistic models associated with clinical studies conducted in human subjects by design integrate these issues. In any case, computational or NHP models should be subjected to an ethical review process before their application to human patients can be considered.

Today DBS research is moving toward closed loop control of disease based on real-time adaptive (intelligent) brain stimulators (Little et al., 2013; McIntyre et al., 2015) using biomarkers, such as local-field potentials. These new approaches are tested in human subjects because there is no alternative able to adapt to the changing behavior of each patient’s brain over time as well as to the evolution of the disease. It should be noted that the novel methods discussed here refer specifically to brain neuromodulation and do not cover disease-modifying treatments, such as drugs, stem cells, and antibodies. Hence, we agree with the concluding remark of the authors (cited above) but the arguments they develop appear somewhat contradictory to their conclusion. As a result, based on historical data and recent developments in the field, we do not support the hypothesis that NHPs constitute a valid scientific modality for the complete understanding of PD and for the development of future neuromodulation therapeutic strategies.

## Author Contributions

AM and AB contributed equally to the commentary.

## Conflict of Interest Statement

Andre Menache receives remuneration from Antidote Europe. Anne Beuter has no conflict of interest to declare.

## References

[B1] Baron M. S. Wichmann T. Ma D. DeLong M. R. (2002). Effects of transient focal inactivation of the basal ganglia in Parkinsonian primates. J. Neurosci. 22, 592–599. 11784807 10.1523/JNEUROSCI.22-02-00592.2002 PMC6758679

[B2] Bechtereva N. P. Bondartchuk A. N. Smirnov V. M. Meliutcheva L. A. Shandurina A. N. (1975). Method of electrostimulation of the deep brain structures in treatment of some chronic diseases. Confin. Neurol. 37, 136–140. 10.1159/000102727 1093777

[B3] Benabid A. L. Pollak P. Louveau A. Henry S. de Rougement J. (1987). Combined (thalamotomy and stimulation) stereotactic surgery of the Vim thalamic nucleus for bilateral Parkinson’s disease. Appl. Neurophysiol. 50, 344–346. 3329873 10.1159/000100803

[B4] Bergman H. Wichmann T. DeLong M. R. (1990). Reversal of experimental parkinsonism by lesions of the subthalamic nucleus. Science 249, 1436–1438. 2402638 10.1126/science.2402638

[B5] Burns R. S. Chiueh C. C. Markey S. P. Ebert M. H. Jakobowitz D. M. Kopin I. J. (1983). A primate model of parkinsonism: selective destruction of dopaminergic neurons in the pars compacta of the substantia nigra. PNAS 80, 4546 10.1073/pnas.80.14.4546 6192438 PMC384076

[B6] DeLong M. R. Benabid A.-L. (2014). Discovery of high-frequency deep brain stimulation for treatment of Parkinson disease: 2014 lasker award. J. Am. Med. Assoc. 312, 1093–1094. 10.1001/jama.2014.11132 25198255

[B7] Drouot X. Oshino S. Jarraya B. Besret L. Kishima H. Remy P. (2004). Functional recovery in a primate model of Parkinson’s disease following motor cortex stimulation. Neuron 44, 769–778. 10.1016/j.neuron.2004.11.023 15572109

[B8] Fox S. H. Brotchie J. M. (2010). The MPTP-lesioned non-human models of Parkinson’s disease. Past, present and future. Prog. Brain Res. 184, 133–157. 10.1016/S0079-6123(10)84007-5 20887873

[B9] Godlee F. (2014). How predictive and productive is animal research? BMJ 2014, g3719 10.1136/bmj.g3719

[B10] Halliday G. Herrero M. T. Murphy K. McCann H. Ros-Bernal F. Barcia C. (2009). No Lewy pathology in monkeys with over 10 years of severe MPTP parkinsonism. Mov. Disord. 24, 1519–1523. 10.1002/mds.22481 19526568

[B11] Jasper H. H. (1966). Recording from microelectrodes in stereotactic surgery for Parkinson’s disease. J. Neurosurg. 24, 219–221.

[B12] Johnston T. M. Fox S. H. (2015). Symptomatic models of Parkinson’s disease and l-DOPA-induced dyskinesia in non-human primates. Curr. Top. Behav. Neurosci. 22, 221–235. 10.1007/7854_2014_352 25158623

[B13] Langston J. W. Ballard P. Tetrud J. W. Irwin I. (1983). Chronic parkinsonism in humans due to a product of meperidine-analog synthesis. Science 219, 979–980. 10.1126/science.6823561 6823561

[B14] Little S. Pogosyan A. Neal S. Zavala B. Zrinzo L. Hariz M. (2013). Adaptive deep brain stimulation in advanced Parkinson disease. Ann. Neurol. 74, 449–457. 10.1002/ana.23951 23852650 PMC3886292

[B15] McIntyre C. C. Chaturvedi A. Shamir R. R. Lempka S. F. (2015). Engineering the next generation of clinical deep brain stimulation technology. Brain Stimulat. 8, 21–26. 10.1016/j.brs.2014.07.039 25161150 PMC4501497

[B16] Modolo J. Legros A. Thomas A. W. Beuter A. (2010). Model-driven therapeutic treatment of neurological disorders: reshaping brain rhythms with neuromodulation. Interface Focus 1, 61–74. 10.1098/refs.2010.0509 22419974 PMC3262241

[B17] Shanks N. Greek R. (2009). Animal Models in Light of Evolution. Boca Raton, FL: Brown Walker.

[B18] Verdier J. Acquatella I. Lautier C. Devau G. Trouche S. Lasbleiz C. (2015). Lessons from the analysis of nonhuman primates for understanding human aging and neurodegenerative diseases. Front. Neurosci. 9:64. 10.3389/fnins.2015.00064 25788873 PMC4349082

